# Mosaic mutations in blood DNA sequence are associated with solid tumor cancers

**DOI:** 10.1038/s41525-017-0025-4

**Published:** 2017-07-06

**Authors:** Mykyta Artomov, Manuel A. Rivas, Giulio Genovese, Mark J. Daly

**Affiliations:** 1 Broad Institute, Cambridge, MA 02139 USA; 2 Analytic and Translational Genetics Unit, MGH, Boston, MA 02114 USA; 3 Department of Chemistry and Chemical Biology, Harvard University, Cambridge, MA 02138 USA; 4 Department of Biomedical Data Science, Stanford University, Stanford, CA 94305 USA

## Abstract

Recent understanding of the causal role of blood-detectable somatic protein-truncating DNA variants in leukemia prompts questions about the generalizability of such observations across cancer types. We used the cancer genome atlas exome sequencing (~8000 samples) to compare 22 different cancer phenotypes with more than 6000 controls using a case–control study design and demonstrate that mosaic protein truncating variants in these genes are also associated with solid-tumor cancers. The absence of these cancer-associated mosaic variants from the tumors themselves suggest these are not themselves tumor drivers. Through analysis of different cancer phenotypes we observe gene-specificity for mosaic mutations. We confirm a specific link between PPM1D and ovarian cancer, consistent with previous reports linking PPM1D to breast and ovarian cancer. Additionally, glioblastoma, melanoma and lung cancers show gene specific burdens of mosaic protein truncating mutations. Taken together, these results extend existing observations and broadly link solid-tumor cancers to somatic blood DNA changes.

## Cancer: Blood mutations linked to solid tumor risk

Increased risk for solid tumors linked to mutations detected in blood samples that shorten the coding sequence of their genes. Mark Daly and colleagues from the Broad Institute in Cambridge, Massachusetts, USA, used large genomic databases to test whether having blood cells both with and without genetic variants predicted to shorten the encoded protein—a phenomenon known as mosaic protein-truncating variants (PTVs)—was associated with developing a range of solid-tumor cancers. They studied DNA from around 8,000 people with cancer and 6,000 healthy controls. Other studies have shown that mosaic PTVs precede and predict the development of leukemia. In this study, the scientists not only confirmed previous reports linking these variants to breast and ovarian cancer but also extended the association to include tumors of the brain, skin and lungs. These results broadly connect cancer to blood DNA changes.

## Introduction

Several recent studies^1, 2^ have reported associations of mosaic protein truncating variants (PTV) in *PPM1D, TET2, ASXL1*, and *DNMT3A* with blood cancers. Intriguingly, such mosaic mutations in *PPM1D* have also been convincingly associated with breast and ovarian cancer^3^—however, since these mutations are somatic, rather than germline, their role in causation has not been clear. We sought to more fully explore the relationship of these somatic mutations, clearly causally linked to blood cancers, in solid tumor cancer using a large assembly of germline and somatic exome DNA sequences of 7979 cancer cases from the cancer genome atlas (TCGA)^4^ and performed a large-scale case–control study with 6177 population controls with no cancer phenotype reported.

## Results

Using data available from dbGAP, we performed a large-scale joint variant calling of sequences generated from blood-derived germline DNA samples from cancer cases and controls—primarily from an assembly of TCGA samples (cases) compared with unselected population controls (with no known cancer status) from several studies (NHLBI-ESP, 1000 Genomes, ATVB, T2D, Ottawa Heart) appropriately consented for broad use as controls. Importantly, all cases and controls in this analysis have age at DNA sampling available (Supplementary Table 1).

Observations of the mosaic mutations might be affected by several parameters—both biological (age,^5^ clinical interventions^6, 7^) and technical (depth of coverage, variant calling accuracy). To make the case–control comparison robust we first identified what adjustments to the model of association are needed.

We observed 348 PTVs (stop gain, essential splice site, frameshift mutations) in the four established somatic leukemia genes. Detection of somatic mutations with low non-reference allele balance is heavily dependent on sequencing depth. To ensure equal sensitivity in cases and controls we first compared coverage of these genes in cancer germline (average 33× coverage) and control (average 29× coverage) data. We next looked specifically at cases and controls carrying PTVs. For germline heterozygous sites the expected allele balance is 0.5, so we applied a binomial test to detect significantly low allele balance genotypes based on depth of coverage and number of alternative reads. Those with *p* < 0.001 (i.e., heterozygotes with significantly <50% non-reference allele) and more than 20× coverage were determined to be mosaic and kept for further analysis (Supplementary Figs. 1 and 2). To further investigate any statistical bias due to sequencing coverage of cases and controls—we tested whether there is any statistical difference in coverage and ref/alt reads counts between cancer cases and controls that carry at least one PTV in the four candidate genes with generalized linear model testing (Supplementary Methods). Cancer status of a sample appears to be non-significant (*p* = 0.279, *p* = 0.898 if adjusted for age) parameter, confirming that called PTVs are adequately covered in both cases and controls and protein-truncating mosaic events have equal chances to be detected in both cohorts. We compared the probability of calling a protein truncating DNA variant in cases and controls with respect to coverage (Suplementary Fig. 3). There is slightly higher sensitivity for the detection of DNA variants in cases, thus we adjusted further analysis for coverage differences (Supplementary Methods). From these analyses, we conclude that all minor technical differences in sensitivity of mosaic variants search in cases and controls were accounted for—a pre-requisite for subsequent analyses.

We then investigated the effect of biological parameters on observation of the mosaic mutations—age and cancer therapy effects. Since our controls were, on average, roughly 10 years younger than the cancer cohort and age has been shown to be a strong predictor of the existence of somatic mosaic mutations, inclusion of age in the association model is critical (Supplementary Methods). Older samples expectedly have higher probability of finding a mosaic variant (Suplementary Fig. 4).^5^ Thus for case-control analysis we adjusted our model for age differences between cases and controls.

Another set of biological parameters to control for is clinical intervention. Specifically, chemotherapy and radiation treatment are of great importance and may alter somatic mutation rates. Within limited available clinical data in TCGA we saw no clear associations to treatment history—neoadjuvant treatment history (*p* = 0.116), radiation therapy (*p* = 0.348), pathologic tumor stage (*p* = 0.354) or other outcome variables when adjusted for age and cancer subtype with mosaic PTV carrier status (Supplementary Tables 2, 3, and 4). This observation is consistent with previous reports of mosaicism in cancer case–control study.^8^ Jacobs et al.^8^ reported no associations to smoking or cancer therapy using genome wide association study (GWAS) arrays, while confirmed associations to age and cancer status. Thus, we did not incorporate clinical parameters into further case–control model.

We then tested the association between mosaic PTV and cancer status by generating a data set consisting of 7979 cancer cases and 6177 controls (Supplementary Methods). We applied a binomial generalized linear model considering age, coverage depth, and mosaic PTV carrier status and found significant evidence of association with cancer status (*p* = 0.00108, odds ratio (OR) = 1.26; OR confidence interval (CI) = 1.1–1.47). Since it was previously shown that *PPM1D* PTVs are associated with breast and ovarian cancers, we removed breast and ovarian cancer samples and repeated the analysis. It confirmed the observed association (*p* = 5.67 × 10^−4^, OR = 1.3; OR CI = 1.12–1.52)—suggesting that reported observations regarding *PPM1D* and breast and ovarian cancers are more general. We also adjusted our model for minor coverage differences between cases and controls (Supplementary Methods, Supplementary Fig. 5, Supplementary Table 5).

It is known that PTVs in the last exon of *PPM1D* specifically that carry ‘‘gain-of-function’’ effect are enriched in cases of breast and ovarian cancer.^1^ We observed the same enrichment in our data set—of 18 mosaic PTVs in *PPM1D*, 17 were in the last exon of the gene. We tested the ‘‘gain-of-function’’ PTV hypothesis in other candidate genes as well (Supplementary Fig. 6). *ASXL1* follows the same pattern as *PPM1D*—35 out of 40 PTVs in this gene are found in the last exon. *TET2* has strong enrichment of exon 3–44 out of 50 PTVs. This is intriguing because *TET2* transcript *ENST00000305737* has three exons and demonstrates enrichment of the last exon. Moreover, this transcript is mostly expressed in whole blood and EBV-transformed lymphocytes according to GTEx database. *DNTM3A* has no known pattern of mosaic PTVs distribution within the gene. Genovese et al.^1^ reported enrichment of the last exons of *DNMT3A* with mosaic missense mutations in leukemia cases. We observed similar enrichment in exons 17–23 (Supplementary Fig. 7). However, no further studies are available to confirm whether missense mutations in this region also have ‘‘gain-of-function’’ effect similar to the other candidate genes.

**Fig. 1:**
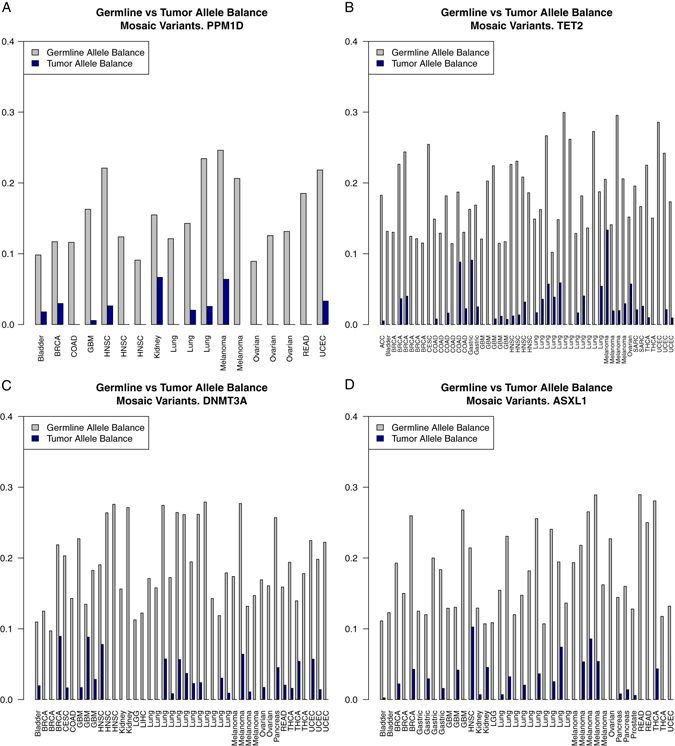
Blood vs. tumor allele balance for each sample with mosaic PTV in **a** PPM1D, **b** TET2, **c** DNMT3A, **d** ASXL1. Observed in blood mosaic mutations are strongly depleted from the tumor somatic DNA (Wilcoxon test *p* < 10^−16^)

As previously demonstrated, mosaic PTVs in the list of candidate genes have been demonstrated to precede and predict the development of leukemia, indicating a causative role.^1, 2, 9–11^ To determine the role of mosaic mutations in solid tumors we evaluated the quantity of mosaic PTVs between tumor and germline DNA in cancer samples. Mosaic PTVs in the candidate genes present in blood DNA were largely absent in tumor DNA from the same individual (Fig. 1). Complete absence of these mutations in tumor sample is impossible due to ineluctable blood contamination of any tumor sample, however, our data strongly indicates that these events in the blood did not represent residual evidence from driver mutations involved in tumor development (in which case we would have expected higher, or perhaps 100% of the mutated allele to be found). As before, we compared coverage in tumor and germline DNA samples and, consistent with the design of TCGA, that tumors have similar or better coverage indicating that the deficit of these mosaic events in tumors is not sensitivity based (Supplementary Fig. 8). This observation is consistent with the findings of mosaic *PPM1D* variants in breast/ovarian cancers.^3^

**Fig. 2:**
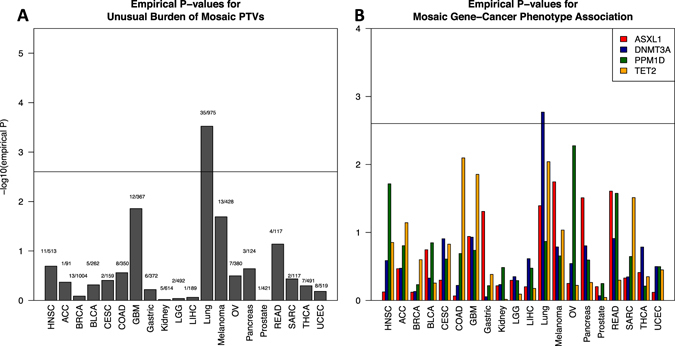
Solid-tumor cancer phenotypes show gene specificity with respect to mosaic PTVs. **a** Empirical enrichment of the different cancer phenotypes with mosaic PTVs **b** Per gene significance of mosaic PTV burden in each cancer phenotype. Experiment-wise significance level is set with Bonferroni correction for multiple phenotypes tested. Ovarian cancer shows previously reported specific association to PPM1D mosaic PTVs

We considered whether presence of mosaic PTVs showed any evidence of cancer specificity. Under the null hypothesis, mosaic PTVs are expected to be found in all candidate genes at the same rate in each of 20 cancer phenotypes. We first tested if any of the cancer phenotypes shows an unusual burden of mosaic PTVs. The empirical significance of observed mosaic PTV frequency deviation from null was assessed using the following scheme: For each cancer phenotype of *N* cases we drew random set of *N* samples from the pool of all cancer cases. Since age strongly affects the frequency of mosaic variants within cohort, only random sets with insignificant (as shown by Wilcoxon test) age difference from the target set were accepted. The empirical *p*-value was then calculated as the fraction of random sample sets with a mosaic PTVs frequency greater than the target set. Statistical significance threshold is given by multiple hypothesis testing correction considering 20 tested phenotypes—0.05/20 (0.0025). (Fig. 2a, Supplementary Methods). Glioblastoma, melanoma, and lung cancers demonstrate a significantly increased burden of mosaic PTVs compared to other cancers. We then examined the distribution of mosaic PTVs across the candidate genes in each cancer phenotype (Fig. 2b, Supplementary Table 6). A similar approach was used as before: for each phenotype we estimated mosaic PTV frequencies in each of the candidate genes. Next, random sets of cancer cases with similar age distribution were generated. For each candidate gene the significance was estimated as fraction of random sets with greater mosaic PTV frequency in a gene of interest. The hypothesis of whether any gene has prevalent burden has been tested in 20 phenotypes, resulting in Bonferroni correction 0.05/20 for statistical significance threshold. It appears that several cancer types show a trend for accumulation of mosaic mutations in specific genes. Intriguingly, ovarian cancer is specifically associated with *PPM1D* mutations, which is supported by previous report.^3^ We also observe associations of head and neck squamous cell carcinoma with *PPM1D*, colorectal adenocarcinoma, and glioblastoma with *TET2*. Interestingly, cutaneous melanoma is associated with *ASXL1* mosaic mutations as *ASXL1* has protein-interaction with *BAP1*, a well-established risk factor for melanoma.^12^ Lung cancer shows a burden of mosaic mutations that is distributed across several genes with *DNMT3A* being the most statistically significant. However, *ASXL1* and *TET2* show a nearly significant trend, suggesting no specificity in accumulation of the mosaic mutations.

We used the previously reported set of samples from Swedish national patient registers^1^ to estimate the frequency of mosaic PTVs and associated solid-tumor cancer development in a population unselected for cancer. Accurate clinical records are available for this cohort so we sought to confirm our statistical approach for TCGA cohort.

We removed from analysis all samples that had an evidence of leukemia or lymphoma developed before the DNA collection as well as those samples that have mosaic missense mutations in *DNMT3A* to estimate the contribution of the PTVs only. The final data set for this analysis consisted of (83 mosaic PTV carriers and 10,867 non-carriers) samples. There were 11 individuals with pre-DNA collection record of the solid-tumor cancer in the cohort of mosaic PTV carriers and 1105 samples with record of solid-tumor cancer among non-carriers. We tried using different thresholds for age of the samples to estimate significance of enrichment. However, due to a small incidence of the mosaic mutations in the population unselected for cancer, this test was inconclusive (Supplementary Table 7A).

We added mosaic missense *DNMT3A* mutations carriers to the mosaic samples cohort and repeated population analysis (Supplementary Table 7B). This resulted in a total of 153 mosaic mutation carriers. There were 26 individuals (~17%) with pre-DNA collection record of the solid-tumor cancer in the cohort of mosaic PTV carriers (1104—about 10% cancer records in 10,870 non-mosaic samples). Once corrected for age this enrichment appears to be insignificant, thus for samples unselected for cancer a much larger cohort is needed to reach a significant conclusion. However, we do observe a trend toward higher incidence of mosaic mutations in samples with cancer history (Supplementary Fig. 9). We analyzed effect of smoking among 4926 samples and saw no enrichment of smokers or former smokers in mosaic carriers (*p* = 0.965 PTVs only, *p* = 0.691 PTVs and mosaic missense in *DNMT3A*, Supplementary Table 8).

Analysis of larger clinical data should provide a clearer answer to whether mosaic mutations are precursors of cancer (and potentially play a causal role) or perhaps are non-causally associated as byproduct of previous therapy for an earlier cancer. Our analyses of these features are power-limited at this point and there is as yet no consensus surrounding this question. While genetic studies suggest that there is no correlation between cancer therapy and burden of mosaic mutations,^8^ clinical reports suggest that chemotherapy is one of the strong drivers of clonal expansion.^6, 7^

## Discussion

Our study investigates the association of the mosaic protein-truncating variants in four genes previously associated with blood cancer risk in blood samples from patients with solid-tumor diagnoses.

We extend the previously observed strong association of mosaic PTVs with increased risk of leukemia to solid-tumor cancers. There are several possible explanations for such an observation. Recent findings in ovarian and breast cancer suggest a significant role of chemotherapy exposure in observed burden of mosaic PTVs in *PPM1D*.^6, 7^ Though our study lacks sufficiently detailed records of chemotherapy treatment to extend those observations, the breadth and robustness of the results here suggest that such an effect of treatment exposure may more generally apply to other candidate genes, cancer phenotypes and specific therapeutics. At the same time analysis of cancer case-control GWAS arrays did not report any association with cancer therapy regimens, or carcinogen exposure (smoking).^8^ While there is no unity in the field on this question, our observations of differences in PTV burden gene specificity according to cancer phenotype suggests that there could be some level of specificity of chemotherapy drugs to cause expansion/survival of certain mutated peripheral blood mononuclear cells clones. Importantly, however, such a link may provide a more general—and detectable—connection between early solid tumor diagnoses and enriched later incidence of leukemia.

There are other possible explanations for the observed association. First, there could be immune system changes in response to early pre-clinical stage of cancer. Our additional screening of early onset cancer cases (breast and ovarian cohort with cancer onset before 35, *N* = 374) shows no enrichment in mosaic PTVs suggesting that this hypothesis is likely irrelevant and age of the samples plays important role (or serving as a trigger) for emergence of clonal expansion. Second, is a potential causal relationship. While a direct role as tumor drivers is ruled out by the absence of PTVs in tumors, we cannot completely eliminate the possibility that these represent a background cancer risk state but find no strong support for this hypothesis. Given fewer than 1% of the population carries a PTV in one of these candidate genes, a large-scale population study with a long-term pre- and post-cancer DNA collection and detailed treatment details will be needed to confidently answer the question whether blood mosaic PTVs are precursors or result of treatment for solid-tumor cancers.

### Data availability

Findings in this manuscript were previously reported on the BioRxiv Preprint server.^13^

Discovery data set included cases from TCGA. All sequencing data is available from dbGAP (accession phs000178.v1.p1). Set of controls included samples from NHLBI Exome Sequencing Project (details could be found http://evs.gs.washington.edu/EVS/), T2D-Genes study (http://www.type2diabetesgenetics.org/projects/t2dGenes), ATVB cohort (dbGAP accession phs000814.v1.p1) and Ottawa Heart study (dbGAP accession phs000806.v1.p1).

Details on availability of the Swedish biobank data set could be found in original publication by Genovese et al.^1^

## Methods

### Data set

Genotypes data set was created by joint variant calling of cancer cases and non-cancer controls using HaplotypeCaller (GATK-3.0)^14–16^ with Broad Institute calling pipeline. For functional annotation of variants we used Variant Effect Predictor by Ensembl.^17^

PCA was performed to keep for analysis only samples of European ancestry to eliminate possible population effects. PCA was performed with EIGENSTRAT.^18, 19^

Resulting genotype file was used to create a PLINK/SEQ (Https://atgu.mgh.harvard.edu/plinkseq/. PLINK/SEQ) project for further manipulations.

### Clinical data

For testing relevance of the mosaic PTVs to medical treatment/outcome clinical data was downloaded from TCGA web-site https://tcga-data.nci.nih.gov/tcga/dataAccessMatrix.htm. All patients provided informed consent for research use of the collected data.

### Generalized linear model and statistical tests

For further statistical tests we used R-3.0.^20^

For further details, please, refer to Supplementary Methods.

## Electronic supplementary material

Supplementary Tables (large file)

Supplementary Materials

## References

[1] Genovese G (2014). Clonal hematopoiesis and blood-cancer risk inferred from blood DNA sequence. N. Engl. J. Med..

[2] Jaiswal S (2014). Age-related clonal hematopoiesis associated with adverse outcomes. N. Engl. J. Med..

[3] Ruark E (2012). Mosaic PPM1D mutations are associated with predisposition to breast and ovarian cancer. Nature.

[4] The results published here are in whole or part based upon data generated by the TCGA Research Network. Available at: http://cancergenome.nih.gov/.

[5] Xie M (2014). Age-related mutations associated with clonal hematopoietic expansion and malignancies. Nat. Med..

[6] Pharoah PDP (2016). *PPM1D* mosaic truncating variants in ovarian cancer cases may be treatment-related somatic mutations. J. Natl Cancer Inst..

[7] Swisher EM (2016). Somatic mosaic mutations in *PPM1D* and *TP53* in the blood of women with ovarian carcinoma. JAMA Oncol.

[8] Jacobs KB (2012). Detectable clonal mosaicism and its relationship to aging and cancer. Nat. Genet..

[9] Ley TJ (2010). *DNMT3A* mutations in acute myeloid leukemia. N. Engl. J. Med..

[10] Delhommeau F (2009). Mutation in *TET2* in myeloid cancers. N. Engl. J. Med..

[11] Gelsi-Boyer V (2009). Mutations of polycomb-associated gene *ASXL1* in myelodysplastic syndromes and chronic myelomonocytic leukaemia. Br. J. Haematol..

[12] Carbone M (2013). BAP1 and cancer. Nat. Rev. Cancer.

[13] Artomov, M., Rivas, M. A., Genovese, G. & Daly, M. J. Mosaic mutations in blood DNA sequence are associated with solid tumor cancers. *bioRxiv*. doi:10.1101/065821 (2016). 10.1038/s41525-017-0025-4 PMC5677955 29263833

[14] DePristo MA (2011). A framework for variation discovery and genotyping using next-generation DNA sequencing data. Nat. Genet..

[15] McKenna A (2010). The genome analysis toolkit: a MapReduce framework for analyzing next-generation DNA sequencing data. Genome Res..

[16] Van der Auwera GA (2013). From FastQ data to high confidence variant calls: the Genome Analysis Toolkit best practices pipeline. Curr. Protoc. Bioinformatics.

[17] McLaren W (2016). The ensembl variant effect predictor. Genome Biol..

[18] Price AL (2006). Principal components analysis corrects for stratification in genome-wide association studies. Nat. Genet..

[19] Patterson N, Price AL, Reich D (2006). Population structure and eigenanalysis. PLoS Genet..

[20] Team, R. C. A Language and Environment for Statistical Computing. (2013).

