# Supplementary material for: Mosaic mutations in blood DNA sequence are associated with solid tumor cancers
Source: NPJ Genom Med. 2017 Jul 6;2:22. doi: 10.1038/s41525-017-0025-4 (PMC5677955; doi:10.1038/s41525-017-0025-4)
Supplement: Supplementary file 2 — Supplementary Materials [file 41525_2017_25_MOESM2_ESM.pdf]

SUPPLEMENTARY MATERIALS

Patient Cohorts 2
Building Linear Model for Case-Control Association 2
Mosaic variant gene – cancer phenotype specificity test 3
Supplementary Figures 5

Patient Cohorts

Primary Discovery Set: We used The Cancer Genome Atlas samples available at the Broad Institute (N=7979) and population controls without known cancer phenotype at the time of DNA collection (N=6177). All of the samples were sequenced at the Broad Institute. Libraries were then prepared for sequencing using a modified version of the manufacturer's suggested protocol, automated on the Agilent Bravo and Hamilton Starlet, followed by sequencing on the Illumina HiSeq 2000. Alignment and variant calling was performed using BWA/GATK/Picard pipeline where all of the samples were processed as a single batch. We removed sites with differential coverage to remove any potential bias between cases and controls.

Swedish Biobank Validation Set: A total of 12,380 Swedish research participants with psychiatric diagnoses were ascertained from the Swedish National Hospital Discharge Register, which captures all inpatient hospitalizations. Controls were randomly selected from population registers. We treated cases and controls as a single cohort for all analyses presented below, as none of the mutational variables analyzed below showed any relationship to psychiatric diagnosis after controlling for other factors such as age and smoking. Research participation and DNA sampling took place from 2005 to 2013. The 12,380 samples collected were sequenced in twelve separate waves. The first wave employed an earlier version of the hybrid-capture procedure (Agilent SureSelect Human All Exon Kit), which targets ~28 million base pairs of the human genome, partitioned in ~160,000 intervals, whereas the samples from the other waves used a newer version (Agilent SureSelect Human All Exon v.2 Kit), which targets ~32 million base pairs of the human genome, partitioned in ~190,000 intervals. The first wave was sequenced using Illumina GAII instruments and the remaining waves were sequenced using Illumina HiSeq 2000 and HiSeq 2500 instruments, with pair ended sequencing reads of 76 base pairs across all waves. Sequencing was performed at the Broad Institute of MIT and Harvard across the period of time from 2010 to 2013 (More details available Genovese et. al., NEJM, 2014).

Building Linear Model for Case-Control Association

Defining mosaic genotypes: We kept only well-covered genotypes for further analysis (DP>=20). We used binomial test on the number of alternative reads for heterozygous sites to detect unusual distribution of reads suggesting a mosaic event. Under the null-hypothesis for heterozygous genotypes 50% of alternative reads are expected. So, using our lower boundary of 20x coverage, that provides minimal adequate statistics for binomial test we expect that only several alternative reads should evidence mosaic event rather than a true heterozygote. So, we required binomial p<=0.001 for true mosaics, which ensures matching with expectation (e.g. 3 alt reads vs 17 reference p= 0.002577).

Controlling for coverage differences: To ensure that distribution of alternative and reference reads in PTV carriers does not depend on the cancer status we set up general linear model: (Ref reads, Alt reads) = $\beta_0 + \beta_1$*Cancer Status. With p=0.279 it appears that cancer cases and controls have similar distribution of the reads in the heterozygous

genotypes at protein-truncating variants. Since age has strong impact on the mosaic status we adjusted the above model for age by performing it in two steps, fitting cancer status to age (Cancer Status = $\beta_0 + \beta_1$*Age) and then fitting the pairs of reference and alternative reads for each het genotype to the residues of the first fitting: (Ref reads, Alt reads) = $\beta'_0 + \beta'_1$*Resid(Cancer Status = $\beta_0 + \beta_1$*Age), adjusted for age $p=0.898$ confirms that there is no technical bias in reads distribution. We estimated probability of detecting a protein-truncating variant in cases and controls (Sup. Fig. 3), that is affected by quality of the DNA samples. Under the null we expect same probability of PTV detection with respect to coverage amongst cases and controls. Despite the difference between cases and controls is very tiny, we still adjusted association model for the mean coverage of the sample.

Controlling for biological parameters effects: We have set of parameters available from clinical data – age at the time of DNA sampling, neoadjuvant therapy and radiation therapy treatment, pathologic tumor stage. We used general linear model to assess significance of their contribution:

1) **Age**: We adjusted the model for coverage similarly to the previous model.
   Mosaic Status = $\beta_0 + \beta_1$*Coverage
   Age = $\beta'_0 + \beta'_1$*Resid(Mosaic Status = $\beta_0 + \beta_1$*Coverage)
2) **Clinical Intervention:** Model was adjusted for both coverage and age in order to ensure no bias is present.
   Mosaic Status = $\beta_0 + \beta_1$*Coverage
   Age = $\beta'_0 + \beta'_1$*Resid(Mosaic Status = $\beta_0 + \beta_1$*Coverage)
   Resid(Age = $\beta'_0 + \beta'_1$*Resid(Mosaic Status = $\beta_0 + \beta_1$*Coverage)) = β''_0+ +β''_1*Neoadjuvant Therapy + β''_2*Radiation Treatment + β''_3*Tumor Stage

Case-Control association model: As we identified to ensure a robust comparison of cancer cases to controls model needs to be adjusted for age and coverage.

Mosaic Status = $\beta_0 + \beta_1$*Coverage
Age = $\beta'_0 + \beta'_1$*Resid(Mosaic Status = $\beta_0 + \beta_1$*Coverage)
Cancer Status = $\beta''_0 + \beta''_1$* Resid(Age = $\beta'_0 + \beta'_1$*Resid(Mosaic Status = β_0 + β_1*Coverage))

Mosaic variant gene – cancer phenotype specificity test

Unusual burden of the mosaic variants in cancer phenotype. Under the null model mosaic PTVs should have no specificity to cancer phenotype. This means, that frequency of the mosaic PTVs observation should be the same among each cancer cohort once accounted for age. Frequency of mosaic PTVs in a certain cancer phenotype cohort needs to be compared against large number of randomized sets of cancer samples that have similar age distribution. To generate the random sets of samples we ran a permutation scheme that ensures the age matching between the target and random cohorts. Example: Let the cancer phenotype A cohort have N=100 samples. We would randomly draw 100 samples out of all

cancer samples in the dataset (N=7979) with age within two standard deviations of mean age in phenotype A cohort. We then ran Mann-Whitney test to confirm similarity of the age distributions between random and target sets of samples. If $p<0.05$ – we rejected this permutation and start over. For each permutation we recorded number of mosaic PTVs observed in random set of samples. Fraction of random sets with greater number of mosaic PTVs than in cohort with phenotype A is determined to be empirical p-value.

Mosaic gene specificity to cancer phenotype. This method is largely similar to the previous section. For each phenotype we estimated mosaic PTV frequencies in each of candidate genes. Next, random sets of cancer cases with similar age distribution were generated. For each candidate gene significance was estimated as fraction of random sets with greater mosaic PTV frequency in a gene of interest. Hypothesis of whether any gene has prevalent burden has been tested in 20 phenotypes, resulting in Bonferroni correction 0.05/20 for statistical significance threshold.

Supplementary Figures

Sup. Fig. 1

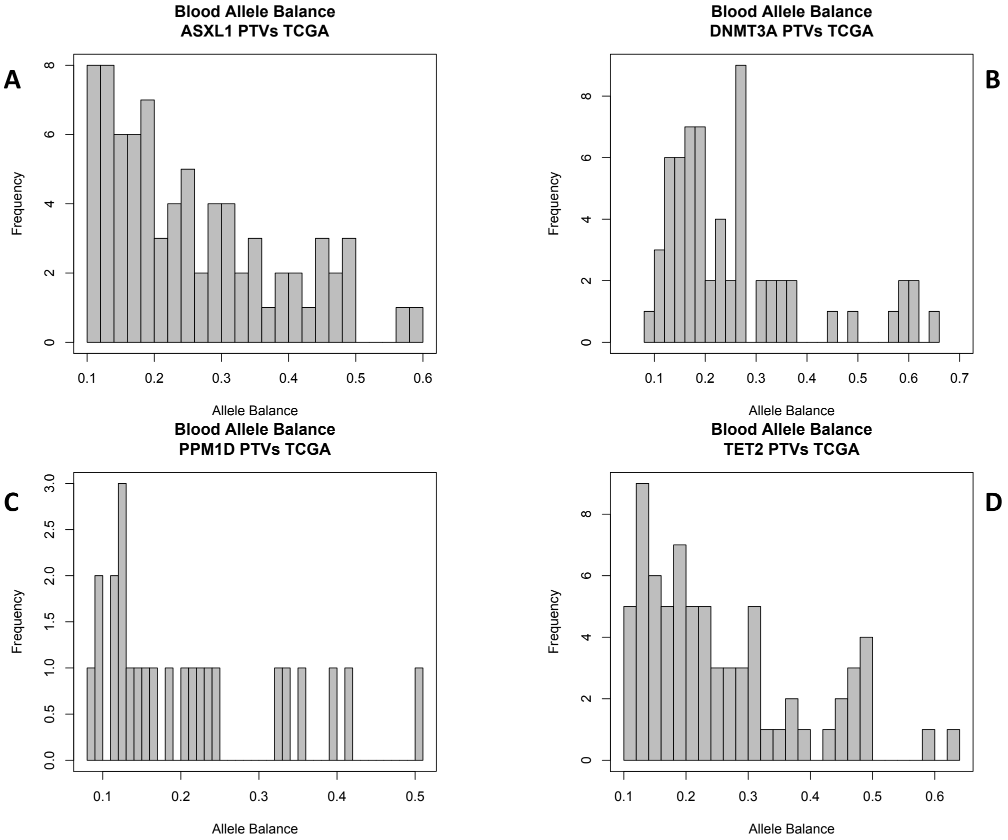

Supplementary Figure 1. Allele balance for all PTVs with >20X coverage in 4 candidate genes in TCGA cancer samples.

Sup. Fig. 2

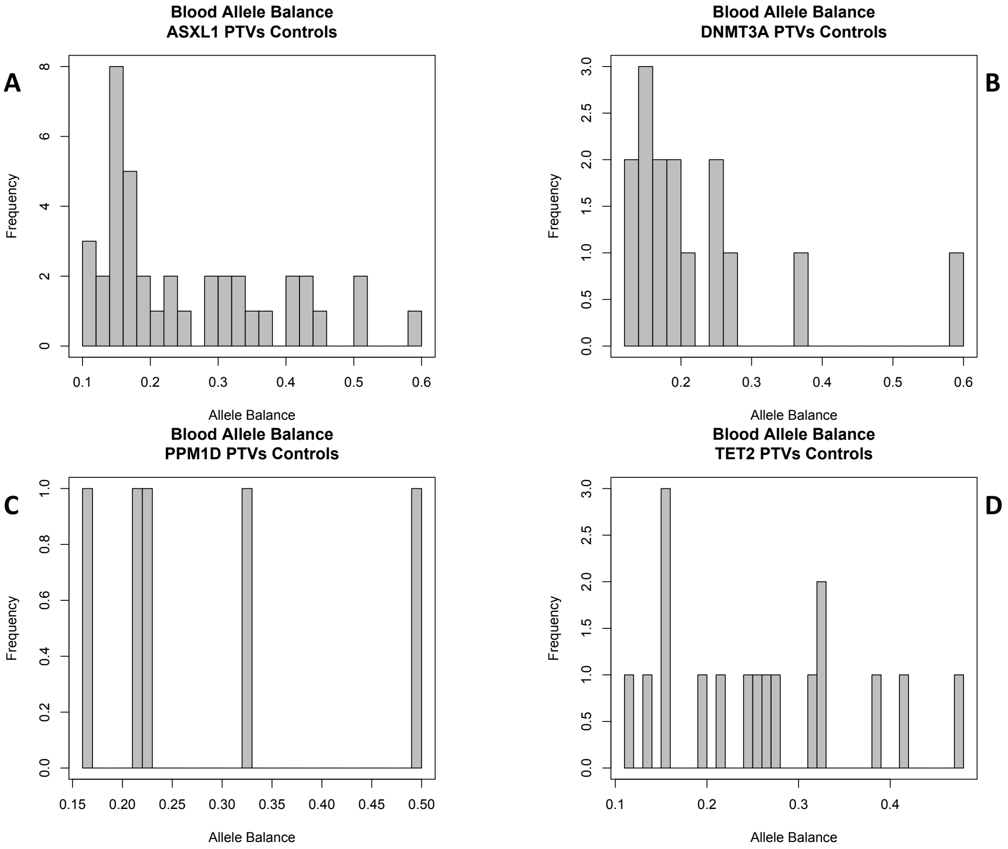

Supplementary Figure 2. Allele balance for all PTVs with >20X coverage in 4 candidate genes in control samples.

Sup. Fig. 3

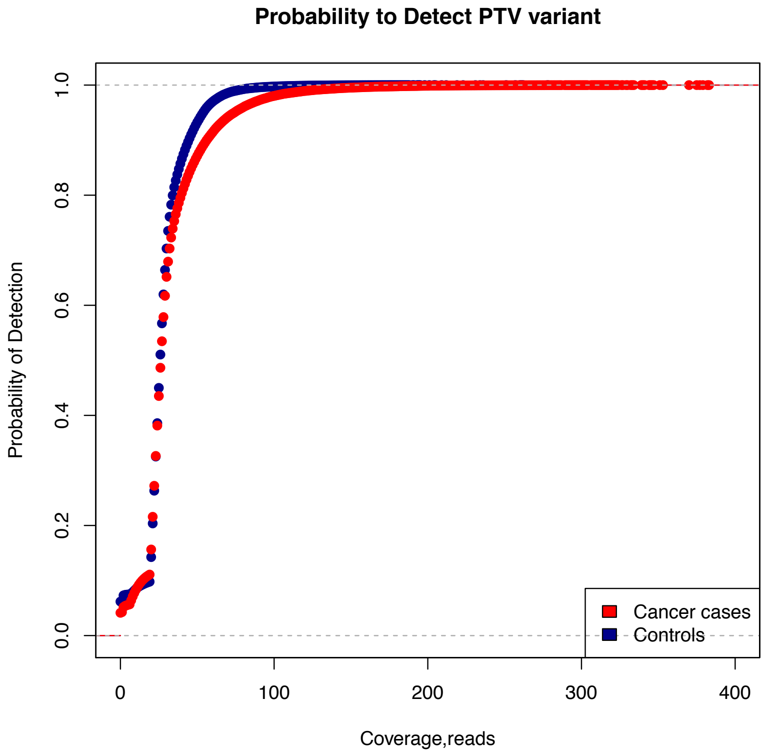

Supplementary Figure 3. Probability of observation a protein-truncating variant in cancer and control samples with respect to coverage.

Sup. Fig. 4

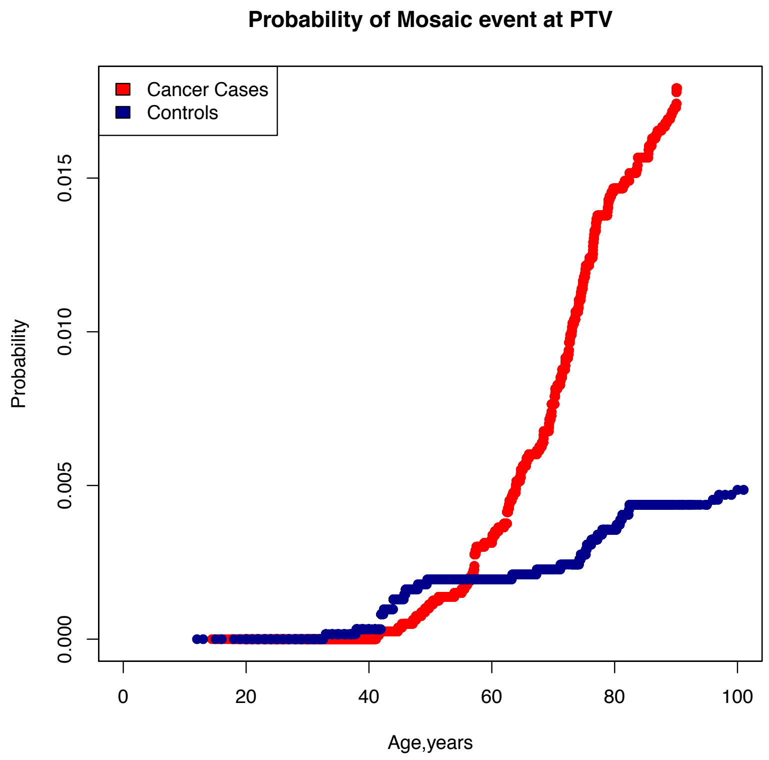

Supplementary Figure 4. Mosaic PTV emergence in blood is strongly correlated with age, however probability of finding such mutations in cancer cases is much greater than in samples with no known cancer history.

Sup. Fig. 5

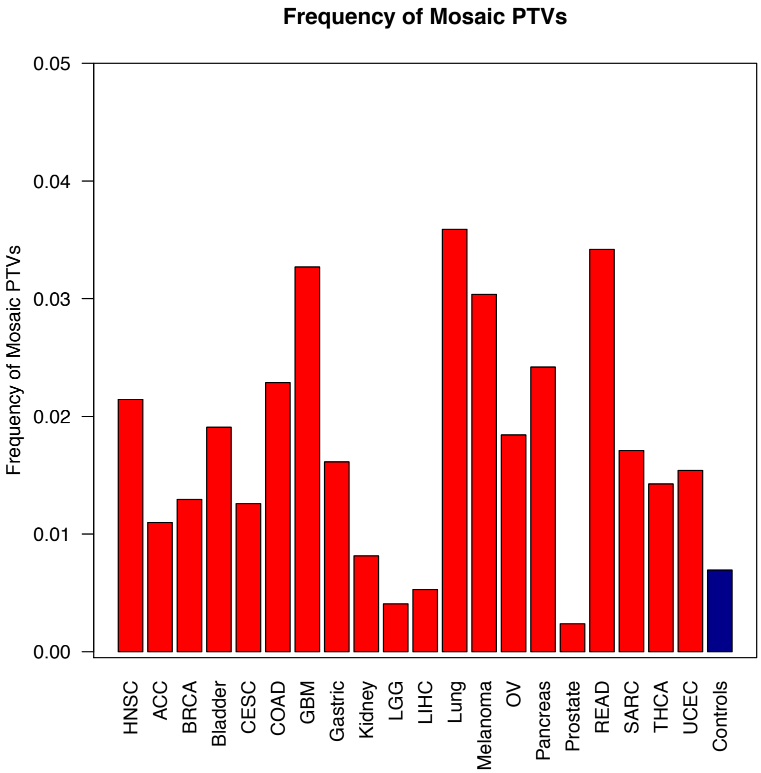

Supplementary Figure 5. Enrichment of the mosaic mutations in cancer cases is observed in almost all cancer phenotypes. Some phenotypes (LGG, LIHC, etc.) show no enrichment either due to the small size of the cohort to detect a substantial amount of mosaic mutations or too young mean age of the samples.

Sup. Fig. 6

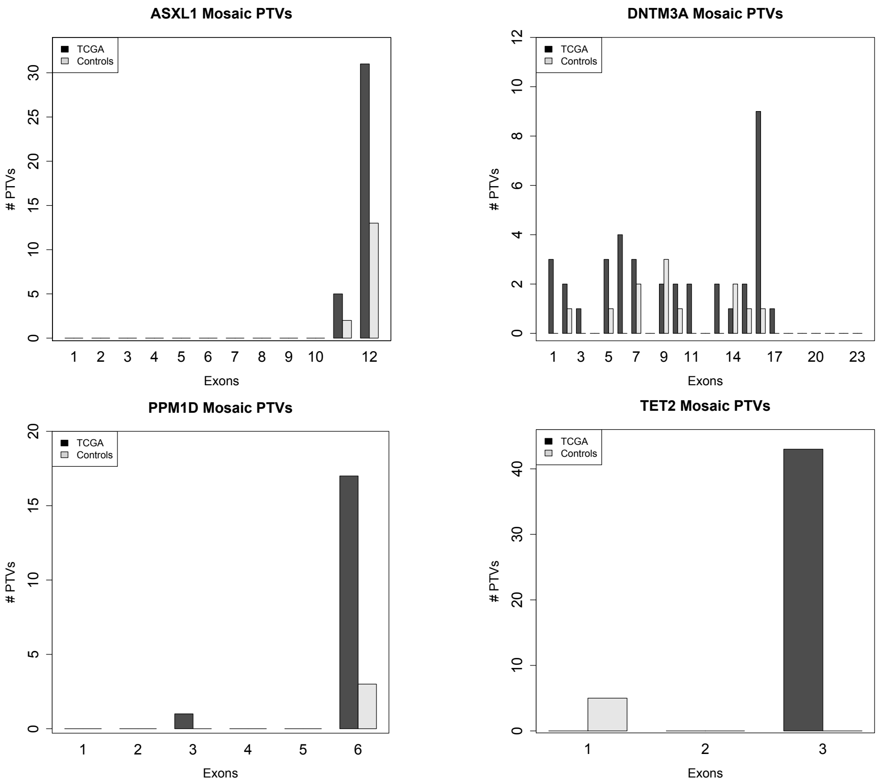

Supplementary Figure 6. PTVs in *ASXL1, TET2, PPM1D* show exon specificity. PTVs in *DNMT3A* shows no specificity. In fact, the last exons of *DNMT3A* are depleted in nonsense variants in both cases and controls.

Sup.
Fig. 7

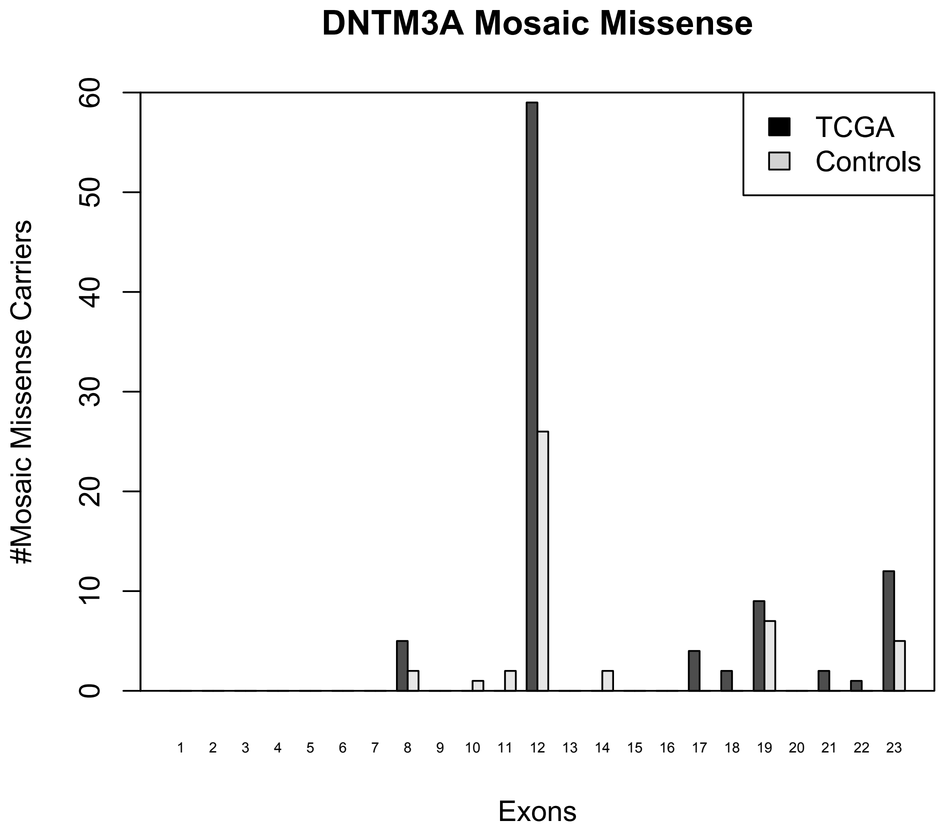

Supplementary Figure 7. Similarly to Genovese et. al. we observe enrichment of the last exons of *DNMT3A* with mosaic missense variants in the blood of both cases and controls.

Sup. Fig. 8

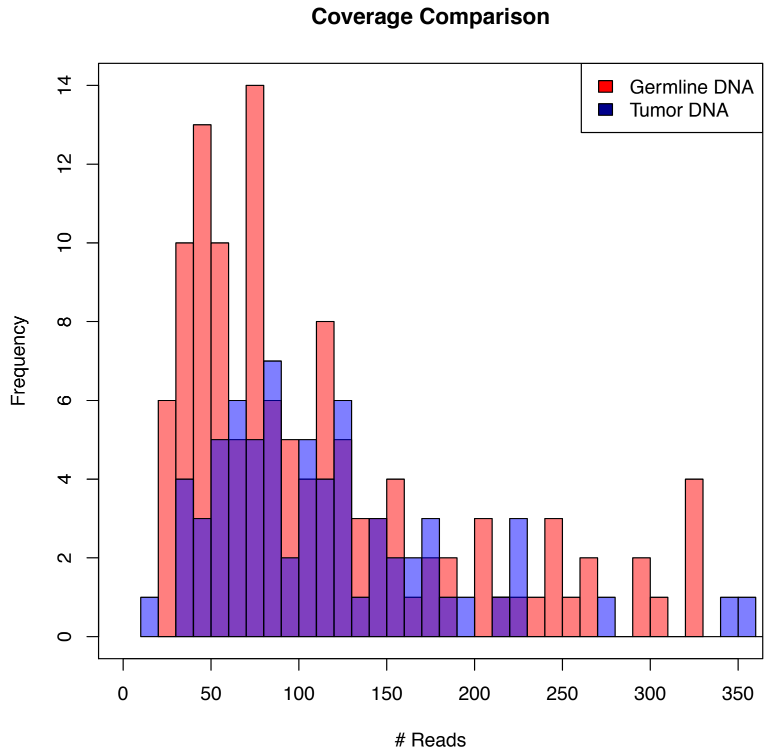

Supplementary Figure 8. Coverage comparison between the TCGA germline and tumor samples. On average tumor DNA has equal or better coverage, than blood DNA.

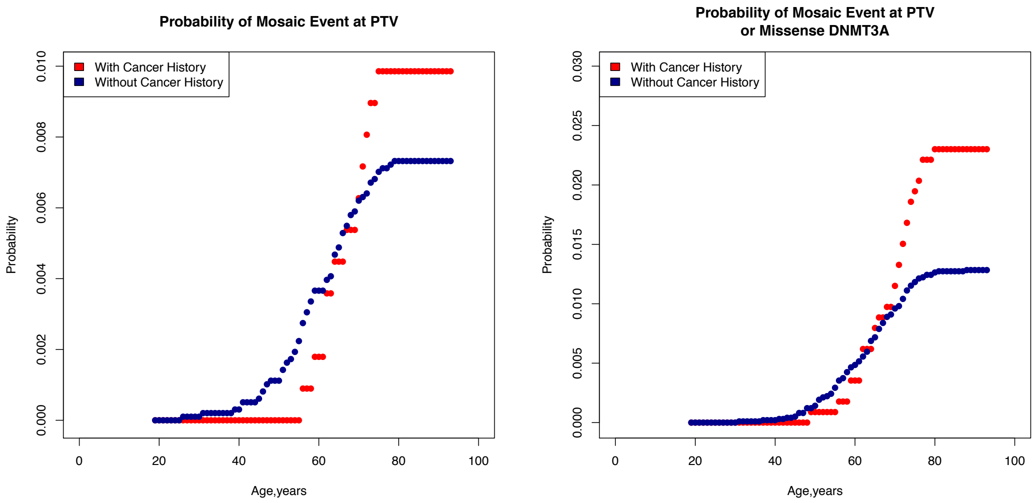

Supplementary Figure 9. Due to low incidence of mosaic mutations in population unascertained for cancer enrichment of the mosaic mutations in the samples with previous history of cancer is not significant in our analysis of Swedish Biobank dataset. However, the trend for higher burden of mosaic mutations in samples with solid tumor cancer history is consistent with findings in our original discovery cohort.
